# Supplementary figures and images for: S. mansoni -derived omega-1 prevents OVA-specific allergic airway inflammation via hampering of cDC2 migration
Source: PLoS Pathog. 2024 Aug 26;20(8):e1012457. doi: 10.1371/journal.ppat.1012457 (PMC11379383; doi:10.1371/journal.ppat.1012457)

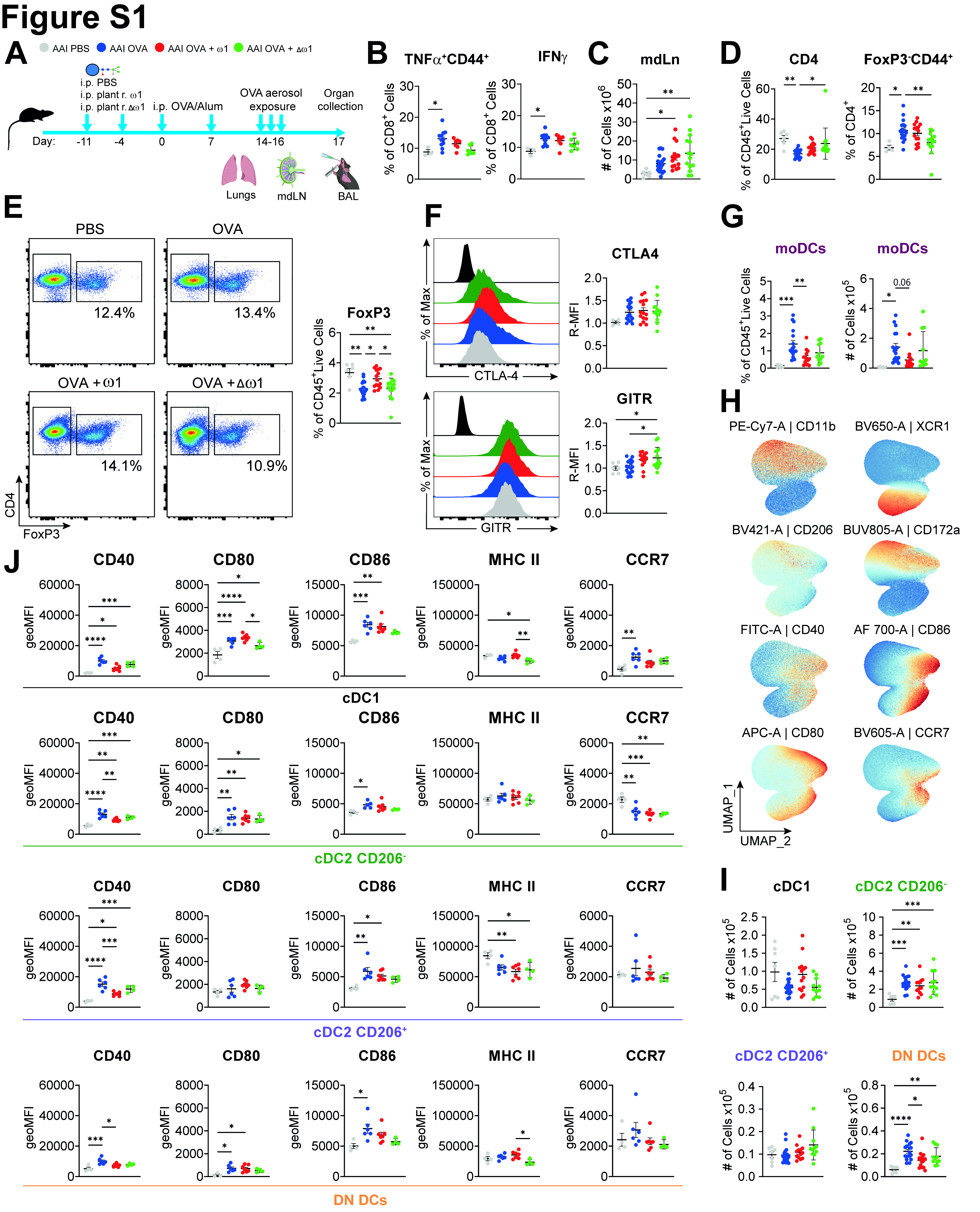

Supplement: S1 Fig — Mice were pre-treated with either PBS, ω1, or mutant ω1 (Δω1) on day -10 and -4. OVA sensitization was induced by OVA/alum on day 0 and 7. Seven days after the last OVA sensitization, mice were challenged for 3 consecutive days by OVA aerosol exposure and sacrificed 24 hours after the last challenge. (A) Schematic view of OVA/alum-induced allergic airway inflammation, created with BioRender.com. (B) Frequency of TNFa+CD44+ (left) and IFNg+CD44+ effector CD8 T cells in the mediastinal lymph node (medLn) of allergic mice after restimulation with PMA and ionomycin in the presence of brefeldin A. (C) Total cell counts of medLn of allergic mice. (D) Frequency of total (left) and FoxP3-CD44+ effector (right) CD4 T cells in the medLn of allergic mice. (E) Representative FACS plot (left) and frequency of CD45+ of regulatory FoxP3+ T cells (right) in the medLn of allergic mice. (F) Representative histograms of CTLA-4 (top left) and GITR (bottom left) and relative geometric mean (R-MFI) of CTLA-4 (top right) and GITR (bottom right) of regulatory FoxP3+ T cell present in the medLn of allergic mice. (G) frequency of CD45+ cells (left) and cell numbers (right) of monocyte-derived dendritic cells present in the lungs of allergic mice. (H) Uniform manifold approximation and projection (UMAP) projections of the lung dendritic cells compartment of allergic mice colored according to the expression of CD11b, XCR1, CD206, CD172a, CD40, CD86, CD80, and CCR7. (I) Cell numbers of DCs subsets identified in the lungs of allergic mice. (J) Geometric median fluorescent intensity (geoMFI) of the expression of CD80, CD86, MHC II, CCR7 and CD40 in lung DCs subsets of allergic mice. Data are pooled from 2 independent experiments with 6 (PBS), 19 (AAI), 15 (AAI + ω1, and 14 (AAI + Δω1) mice (C-G; I) or representative of 1 out 2 independent experiments with 3 (PBS), 11 (AAI), 7 (AAI + ω1, and 7 (AAI + Δω1) mice (B;H; J). Mean ± SEM are indicated in the graphs. One-way Anova with Tukey HSD post-test [file ppat.1012457.s001.tif]

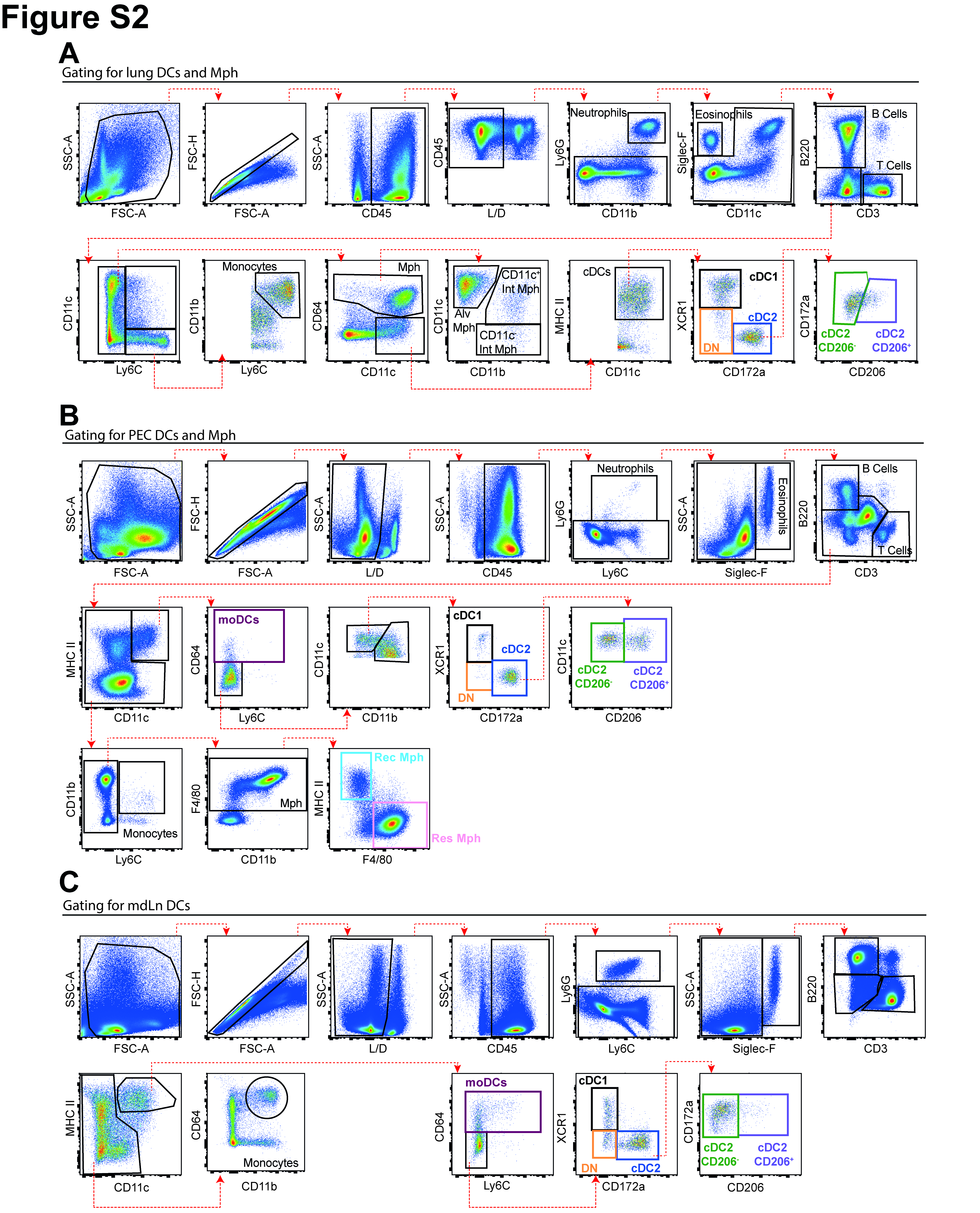

Supplement: S2 Fig — Representative staining and gating strategies for (A) cDC1, CD206+ and CD206- cDC2, double negative (DN) DCs, alveolar (Alv Mph) and interstitial macrophages (Int Mph) in the lungs; (B) cDC1, CD206+ and CD206- cDC2, DN DCs, moDCs resident (Res Mph) and recruited macrophages (Rec Mph) in the peritoneal exudate cells (PEC); (C) cDC1, CD206+ and CD206- cDC2, DN DCs and moDCs in the draining mediastinal lymph node (medLN). (PDF) [file ppat.1012457.s002.tif]

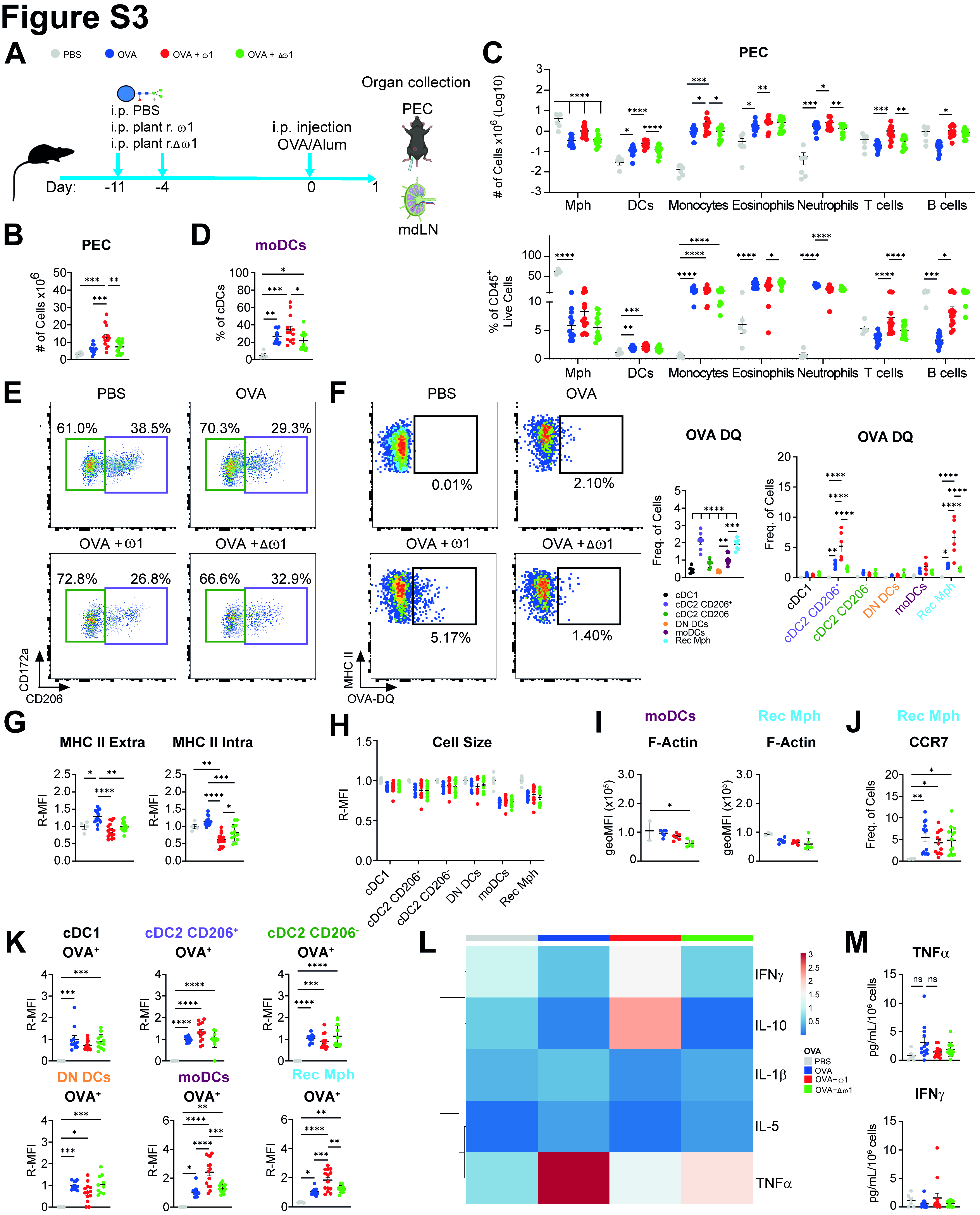

Supplement: S3 Fig — Mice were pre-treated with either PBS, ω1, or mutant ω1 (Δω1) at day 0 and day 7 and 4 days later were exposed to OVA/alum and sacrificed after 24 hours. Peritoneal exudate cells (PEC), lung and medLn were collected. (A) Schematic view of OVA/alum-AAI, created with BioRender.com. (B) Total cell counts of PEC from mice either pre-treated with ω1 or Δω1. (C) Cell number (top panel) and frequency of live CD45+ cell (bottom panel) from different immune cells in the PEC of OVA/alum-treated mice. (D) Frequency of CD45+ cells from monocyte-derived dendritic cells (moDCs). (E) Representative FACS plot of CD206+ and CD206- cDC2 in the PEC of OVA/alum-treated mice. (F) Representative FACS plot (left) and quantification of OVA-DQ processing in the main myeloid cells in the PEC of OVA/alum-treated mice (middle chart) and in the PEC of mice treated with either ω1 or Δω1 (right chart). (G) Relative MHCII in the intracellular and extracellular compartment. (H) Relative MFI (R-MFI) normalized by PBS-treated mice for the quantification of DCs cell size using flow cytometer forward size scatter (FSC) as a parameter. (I) geoMFI of phalloidin, as a readout for filamentous actin (F-Actin), in moDCs and Rec MPh from the PEC of OVA/alum treated mice. (J) Frequency of CCR7-expressing recruited macrophages (Rec Mph) in OVA/alum treated mice. (K) Mice were injected with a mixture of OVA-Alexa Fluor 647 (AF647) and alum and 24 hours later cells from the peritoneal cavity were evaluated for the positivity of OVA. R-MFI of OVA-AF647, inside of OVA+ cells, in DCs, moDCs and Rec Mph in the PEC of OVA-AF647/alum-treated mice. (L) Heatmap displaying the average levels of cytokines adjusted by cell counts in the PEC of OVA/alum-injected mice either treated or not with ω1 or Δω1. (M) Bar graphs of IL-10 and IFNg adjusted by cell counts in the PEC of OVA/alum-injected mice either treated or not with ω1 or Δω1 Data are from one experiment with 3 (PBS), 7 (OVA), 7 (OVA + ω1), and 7 (OVA + Δω1) mice (F, [file ppat.1012457.s003.tif]

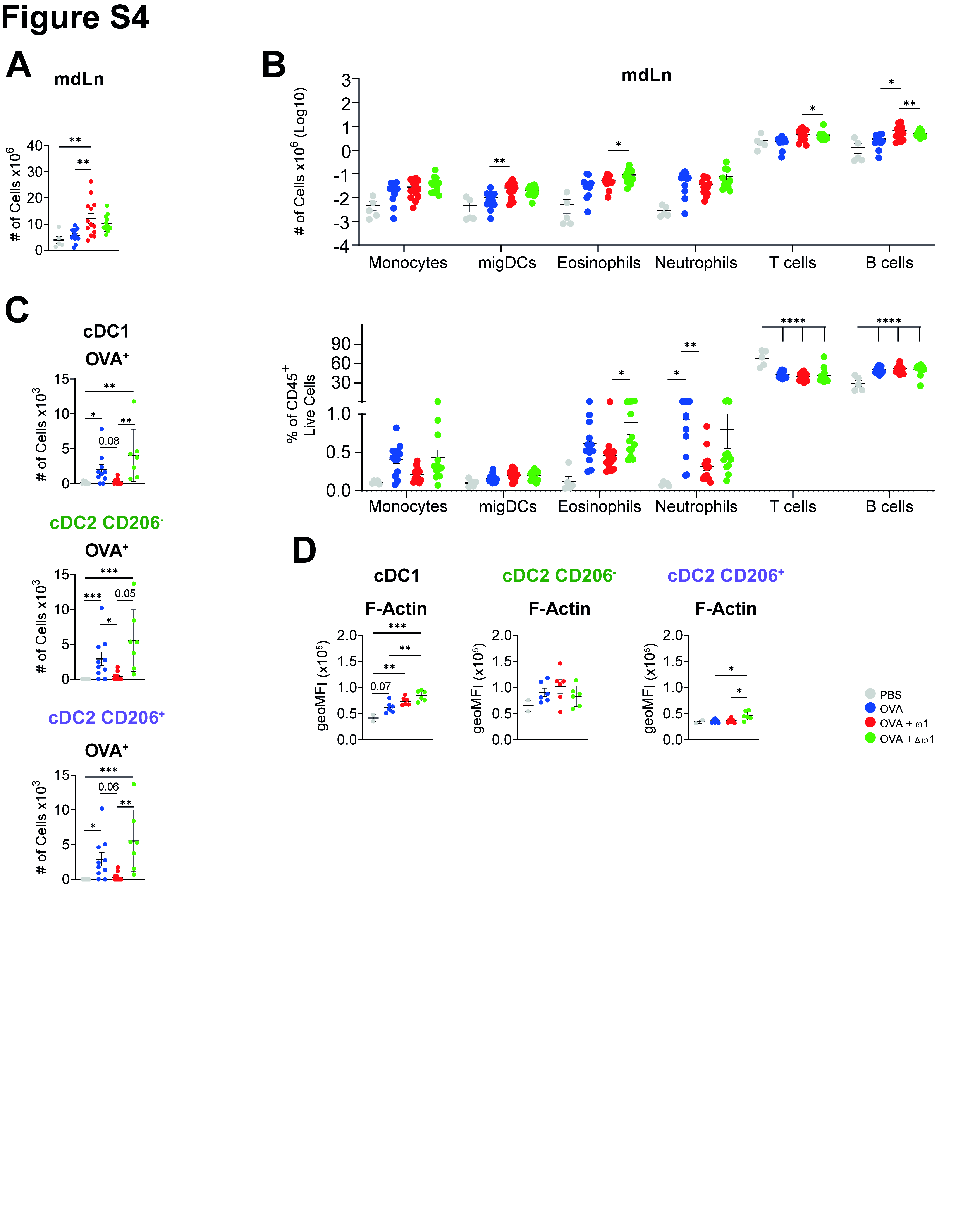

Supplement: S4 Fig — Mice were pre-treated with either PBS, ω1, or mutant ω1 (Δω1) at day 0 and day 7 and 4 days later were exposed to OVA/alum and sacrificed after 24 hours. PEC, lung and medLn were collected. (A) Total cell counts from mdLn of OVA/alum treated mice. (B) Cell number (top panel) and frequency of live CD45+ cell (bottom panel) from different immune cells in the medLN of OVA/alum-treated mice. (C) geoMFI of phalloidin, as a readout for filamentous actin (F-Actin), in DCs from the medLN of OVA/alum treated mice. 2 independent experiments with 6 (PBS), 13 (OVA), 13 (OVA + ω1), and 13 (OVA + Δω1) mice (A-B, D) or 2 independent experiments with 7 (PBS), 10 (OVA), 10 (OVA + ω1), and 7 (OVA + Δω1) mice (C). One-way Anova with Tukey HSD post-test was used to assess statistically significant differences (A-D); *p < 0.05, **p < 0.01, ***p < 0.001, ****p<0.0001. (PDF) [file ppat.1012457.s004.tif]
