# Supplementary material for: S. mansoni -derived omega-1 prevents OVA-specific allergic airway inflammation via hampering of cDC2 migration
Source: PLoS Pathog. 2024 Aug 26;20(8):e1012457. doi: 10.1371/journal.ppat.1012457 (PMC11379383; doi:10.1371/journal.ppat.1012457)
Supplement: S1 Table — List of antibodies used in the current manuscript. (DOCX) [file ppat.1012457.s005.docx]

**Supplemental Table 1: Antibody list**

| Antibodies | Source | Identifier |
| --- | --- | --- |
| Anti-mouse CD11b (Clone M1/70 ) | Invitrogen/eBioscience | Cat# 25-0112-82 |
| Anti-mouse CD11c (Clone N418 ) | BioLegend | Cat# 117330 |
| Anti-mouse CD172a (Clone P84 ) | BioLegend | Cat# 144011 |
| Anti-mouse CD103 (Clone 2E7 ) | BD Biosciences | Cat# 750718 |
| Anti-mouse CD103 (Clone 2E7 ) | Invitrogen/eBioscience | Cat# 13-1031-85 |
| Anti-mouse CD3 (Clone 17A2 ) | Invitrogen/eBioscience | Cat# 11-0032-82 |
| Anti-mouse B220 (Clone RA3-6B2 ) | BioLegend | Cat# 103248 |
| Anti-mouse B220 (Clone RA3-6B2 ) | BD Bioscience | Cat# 553091 |
| Anti-mouse CD3 (Clone 17A2 ) | Invitrogen/eBioscience | Cat# 48-0032-82 |
| Anti-mouse CD3 (Clone 17A2 ) | BioLegend | Cat# 100237 |
| Anti-mouse CD4 (Clone GK1.5 ) | Invitrogen/eBioscience | Cat# 25-0041-81 |
| Anti-mouse CD4 (Clone GK1.5 ) | Invitrogen/eBioscience | Cat# 46-0041-82 |
| Anti-mouse CD4 (Clone GK1.5 ) | BD Biosciences | Cat# 563232 |
| Anti-mouse CD8a (Clone 53-6.7 ) | Tonbo Biosciences | Cat# 20-0081-U025 |
| Anti-mouse CD8a (Clone 53-6.7 ) | BioLegend | Cat# 100714 |
| Anti-mouse CD25 (Clone PC61.5 ) | BD Bioscience | Cat# 551071 |
| Anti-mouse CD25 (Clone PC61.5 ) | BioLegend | Cat# 102010 |
| Anti-mouse CD40 (Clone HM40-3 ) | Invitrogen/eBioscience | Cat# 11-0402-82 |
| Anti-mouse CD44 (Clone IM7 ) | Invitrogen/eBioscience | Cat# 48-0441-82 |
| Anti-mouse CD44 (Clone IM7 ) | Invitrogen/eBioscience | Cat# 25-0441-81 |
| Anti-mouse CD45 (Clone 30-F11 ) | BD Bioscience | Cat# 748370 |
| Anti-mouse CD45 (Clone 30-F11 ) | BD Bioscience | Cat# 564225 |
| Anti-mouse CD45 (Clone 30-F11 ) | BioLegend | Cat# 103173 |
| Anti-mouse CD62L (Clone MEL-14 ) | Invitrogen/eBioscience | Cat# 47-0621-82 |
| Anti-mouse CD8a (Clone 53-6.7 ) | BioLegend | Cat# 100747 |
| Anti-mouse CD64 (Clone X54-5/7.1 ) | BioLegend | Cat# 139304 |
| Anti-mouse CD64 (Clone X54-5/7.1 ) | BioLegend | Cat# 139320 |
| Anti-mouse CD80 (Clone 16-10A1 ) | BioLegend | Cat# 104714 |
| Anti-mouse CD86 (Clone GL-1 ) | BD Bioscience | Cat# 560581 |
| Anti-mouse CD152 (CTLA-4) (Clone UC10-4B9 ) | BD Bioscience | Cat# 12-1522-82 |
| Anti-mouse CD152 (CTLA-4) (Clone UC10-4B9 ) | BioLegend | Cat# 106312 |
| Anti-mouse CD197 (CCR7) (Clone 4B12 ) | BioLegend | Cat# 120125 |
| Anti-mouse CD357 (GITR) (Clone YGITR 765 ) | BioLegend | Cat# 120222 |
| Anti-mouse FoxP3 (Clone FJK-16s ) | Invitrogen/eBioscience | Cat# 12-5773-82 |
| Anti-mouse FoxP3 (Clone FJK-16s ) | Invitrogen/eBioscience | Cat# 17-577382 |
| Anti-mouse IFNγ (Clone XMG1.2 ) | Invitrogen/eBioscience | Cat# 25-7311-82 |
| Anti-mouse IFNγ (Clone XMG1.2 ) | Invitrogen/eBioscience | Cat# 11-7311-41 |
| Anti-mouse IL-10 (Clone JES5-16E3 ) | Invitrogen/eBioscience | Cat# 12-7101-82 |
| Anti-mouse IL-17A (Clone eBio17B7 ) | Invitrogen/eBioscience | Cat# 45-7177-80 |
| Anti-mouse IL-4 (Clone 11B11 ) | Invitrogen/eBioscience | Cat# 17-7041-82 |
| Anti-mouse TNF-a (Clone MP6-XT22 ) | BioLegend | Cat# 506324 |
| Anti-mouse F4/80 (Clone BM8 ) | Invitrogen/eBioscience | Cat# 47-4801-82 |
| Anti-mouse MHCII (Clone M5/114.15.2 ) | Invitrogen/eBioscience | Cat# 56-5321-80 |
| Anti-mouse MHCII (Clone M5/114.15.2 ) | Invitrogen/eBioscience | Cat# 47-5321-82 |
| Anti-mouse MHC II (Clone 2G9 ) | BD Bioscience | Cat# 743876 |
| Anti-mouse Ly6C (Clone HK1.4 ) | BioLegend | Cat# 128025 |
| Anti-mouse Ly6C (Clone HK1.4 ) | Invitrogen/eBioscience | Cat# 45-5932-80 |
| Anti-mouse Ly6G (Clone 1A8 ) | BioLegend | Cat# 108467 |
| Anti-mouse CX3CR1 (Clone SA011F11 ) | BioLegend | Cat# 149025 |
| Anti-mouse Siglec-F (Clone E50-2440 ) | BD Biosciences | Cat# 740388 |
| Anti-mouse Siglec-H (Clone 440c ) | BD Biosciences | Cat# 747675 |
| Anti-mouse XCR1 (Clone ZET ) | BioLegend | Cat# 148220 |
