# Supplementary material for: S. mansoni -derived omega-1 prevents OVA-specific allergic airway inflammation via hampering of cDC2 migration
Source: PLoS Pathog. 2024 Aug 26;20(8):e1012457. doi: 10.1371/journal.ppat.1012457 (PMC11379383; doi:10.1371/journal.ppat.1012457)
Supplement: Attachment — Submitted filename: Plos Pathogens_Response to Reviewers - v2.docx [file ppat.1012457.s007.docx]

**Reviewer #1:**

The manuscript number PPATHOGENS-D-23-01084 reported the host immune response from one of the major proteins from mature Schistosoma mansoni egg secretory product (ESP) named omega-1 to prevent OVA-specific allergic airway inflammation. The authors used either wild type and mutant omega-1 (lack of its ribonuclease activity) recombinant proteins to induce mouse immune cells in vivo prior to expose to OVA immunogen, followed by investigating of secretory cytokines in circulation, intersted tissue immune cell populations and their secretions in vitro. The concept of study and the outcome would be great and fullfill the knowledage about this protein which have been reported as important ESP to egg-induced granulomas in mammalian host. However, the current manuscript has not reached the satisfaction to be accepted for publication yet. The manuscript would be suitable for publications after authors provide more information in the manuscript text especially the results, materials, and method sections. The complexity of experimental setting staring from mouse immune induction by wild type or mutant omega-1 proteins, I would recomment to add the schematic of the experiments cluding mouse immune IP, tissues, and blood sample collection til the down stream experiment ex vivo/in vitro. I am strontly to improve the writing to clarify unclear text especially in Results and Materials and Method sections.

We appreciate the reviewer’s comments, and apologize if, any parts of our results and methods section were not clear. We tried to address all the reviewer’s comments and incorporate as many suggestions as possible , including updates in the suggested schematic figures (Figure S1A and Fig S3A) and rewriting of the indicated sections.

*Required experiment*

While the authors analyzed several cellular parameters as read-out of AAI, I believe that a major one is missing: a functional test of lung capacity (e.g. Penh measurements 24 hours after the final airway challenge using a Buxco system with mice exposed to increasing doses of methacholine or a similar technique). This would be important before proposing a new therapeutic approach based on the observations made in this manuscript.

We appreciate the reviewer’s suggestion, and we have performed a functional test of lung capacity with FlexiVent. Results are now displayed in Figure 1J. The functional data of resistance and elastance corroborates the cellular parameters we had previously observed. We have incorporated these data in the results section (line 107).

*Comments*

*Result section*

Is lack of matric and statistic information in manuscript text. This section needs to be improved to clarify allf of the matric data of individual panel of each figure, along with the value of statistic analysis and number of samples used for statistic analysis.

We appreciate the reviewer’s suggestion on matric and statistical analysis. This information is indeed important to value the quality of the data, but providing all information without restrictions may also risk making the result section more difficult to read and digest. Given the earlier comment by this reviewer on the readability of the result section, we are hesitant to add all this information in the text. Therefore, we decided to use asterisks to depict significancy, and in the figure legends we explain the level of significance provided by *, **, *** or **** levels. Furthermore, each dot in the figures represent an individual mouse, and information about the number of experiments and number of mice is now provided in the figure legends.

Figure legends are insufficient information of the explanation of all the graph such as x- and y-axis, color code used especiall heat maps

We appreciate the reviewer’s comment and understand the complexity of the figures in particular regards heatmaps. To make the graphs more intuitively, we have added the explanation for the y-axis directly in each individual graph from every figure. The x-axis represents the different groups used in the analysis, and each group is color coded in each figure (shown in the top left). We have added the information on the color code of the groups also to the legend. We have synchronized the color coding in the figures as much as possible. In each of the heatmaps the color code for the groups and/or cell type used in the analysis are explicitly named.

No scale bar(s) in panel I of figure 1.

We apologize for the small font size and poor readability. We have now increased the scale bar in the image. Additionally, in the figure legend the size of the scale bar is described (100 µm).

*Material and Methods section*

- Consider adding the schematic of the experimental set up, since there were complex experimental study staring from mouse immune induction through the ex vivo/in vitro experiments

We thank the reviewer for the good suggestion and understand our experiments are complex and need a better graphical explanation. We have updated the schematic representations for the setup of our the experiments, and hope it is more clear now. Moreover, we have updated the methods section to take into accounts reviewer’s comments to make it more structured and clear.

- There was several unclear information as list below, and lack of references of techniques used in this study.

• Lack of H58F-omega-1 sequence information e.g. GenBank accession number or other public sequence submission

The H58F-omega-1 mutant has not been deposited in GenBank, but WT omega-1 is in GenBank under the accession number ABB73003.1: this information has now been added to the methods section, line 325. H58F means that a histidine residue (H) in the T2 RNAse catalytic domain was replaced by phenylalanine (F), resulting in loss of RNAse activity. The amino acid sequence of the WT omega-1 and the generation of the mutant has been already published (22966004), and we emphasized this now in the methods section (line 363).

• First used abbreviations were not clarified, for example, SPF (line 331), LUMC (line 331), OVA (line 334), medLn (line 344), UMAP (line 367).

We appreciate the comments and have added the clarification for SPF (line 370), LUMC (line 7), OVA (line 26), medLN (line 32), and UMAP (line 434).

• ‘in vivo expeiments’ is confusion, the author indicated that mice were pre-treated with omega-1 protein, but there is no explanation of how the authors proceeded for pre-treatment. Was it IP injection? The time of IP injection of OVA and mouse sacrification is unclear.

The pre-treatment with omega-1 was done by IP injection; this is now included in the methods (line 375). Two sets of experiments were performed with IP injection of OVA. In the first set of experiments (induction of allergic airway inflammation), OVA was injected via I.P at day 0 and at day 7 followed by OVA challenge by intranasal exposure at day 14, 15 and 16. Mice were then sacrificed at day 17, 24 hours after the last OVA challenge (The updated scheme is shown in Figure Supplementary 1A). In the second set of experiments (Ova sensitization), OVA was injected via I.P. at day 0 and at day 7, and mice were sacrificed 24 hours after the last OVA I.P. injection (the updated scheme is shown in Figure Supplementary 3A). We included in the methods section a better explanation about the two protocols (line 374).

• ‘Tissue preparation’; the methodology of this sub-section must be provided. It is unclear that after tissue enzymatic digestion, how blood cells need to be lysed (remaining blood cells on lines 346-247). Was it meant collagenase fail to complete to lyse the blood cells? Did tissues mince before enzymatic digestion?

We re-wrote this section in the manuscript to better clarify how single cell suspensions were obtained from the different tissues. Tissue digestion with collagenase is designed to cleave the bond between a neutral amino acid (X) and glycin, abundant in collagen, thus it is able to break the peptide bonds in collagen, facilitating extracellular matrix digestion and releasing cells into suspension (6282982). This process is not intended to kill or lyse the immune (or red blood) cells that are present in the tissue. As consequence, after digestion of blood-enriched tissues (such as the lung and the spleen) the remaining red blood cells are lysed with a specific buffer containing ammonium chloride. Because red blood cells are permeable to this salt, a final consequence is the lysis of red blood cells, but not the immune cells (18083194).

• ‘High dimentional spectral flow cytometry analysis’; what kind of samples (line 365) used in this study? Did they the raw data from flow cytometry?

The samples used for this analysis were the .fcs files, which are the raw files obtained after measuring the samples in a flow cytometer. After importing these samples into the OMIQ software, samples were gated on live CD64^-^CD11c^+^MHCII^+^ lung DCs and analysis were performed as described in the methods section. We adjusted this information in the methods section.

• ‘Migration’; what was migration? Cell migration?, What kind of the medium used(line 376)? What was the diluent of CCI19? What is the detact buffer? Please clarify feeder tray and detach tray and strongly to include the reference(s).

We apologize if the description of this section was unclear. Briefly, the medium used was serum-free RPMI and this was also used to dilute CCL19. We changed the name “detach buffer” to “Cell Detachment Solution”, which is the commercial name from the buffer used to detach the cells from bottom of the membrane. Because this is a commercial kit, we don’t have information about its composition. Feeder tray is the name given by the company to the lower chamber from the migration plate. We change the name “detach tray” to “Cell Harvesting Tray”, which is the commercial name from a second tray used to collect the cells that had migrated but were still bounded to the bottom part of the membrane. We re-wrote this section to make it clearer (line 439).

• ‘T cell restimulation’; it is unclear what kind of cell(s) used in this study. Only Tcell or both DC and Tcell? Please provide more information including reference(s)

We again apologize the reviewer if this section was not clear enough. After isolation of lung immune cells, the single cell suspension is plated in 96 round bottom well plate and stimulate with PMA and Ionomycin in the presence of Brefeldin A for 4 hours. Brefeldin A is an inhibitor of intracellular protein transport, preventing the secretion of cytokines produced after the stimulation. After the incubation time, the cells are stained a viability dye, fixed, permeabilized and stained for antibodies targeting T cells and cytokines. This ensures the identification of which immune cell is producing the cytokine we are interested. We re-wrote this methods section to make it clearer (line 454).

• ‘cytokine maesurment’; authors indicated that the IL-5, IL-10, IL-13 and IFN-g concentration were determined, followed by flow cytometry. Did author meant those secreted cytokines measured from serum and/or tissue culture medium? How flow cytometry used for cytokine measurement?

We attempted to better clarify the material used in the “cytokine measurement” section (now renamed to “cytokine measurement by cytometric bead array”. For this analysis we used the BAL fluid collected from mice and the supernatant of cells isolated from the mediastinal lymph node restimulated with either OVA or omega-1. The cytometric bead array (CBA) is flow cytometry-based method in which multiple soluble cytokines can be evaluated at the same time in culture supernatant or fluid. The sample (BAL fluid or supernatant) is incubated with beads coated with antibodies targeting different cytokines (in our case four beads were used, coated with antibodies against IFNg, IL-5, IL-10 and IL-13). Each bead has a unique fluorescence intensity, allowing us to identify all four different beads in a flow cytometer. During the sample incubation step, a secondary antibody (specific for each analyte) conjugated with PE is used, and the intensity of PE is proportional to the cytokine concentration. This flow cytometry-based assay has been used by now in many publications, and more information can be found in the company website (https://www.bdbiosciences.com/en-nl/products/reagents/immunoassays/cba).

• ‘IgG1 and IgE measurement’; did authors meant sandwich ELISA that enzymatic plate coated with IgG and IgE, the probed with OVA, then react with its antibody before conjugated-antibody kick in? How these antibodies specific to OVA? Antibody enrichment? Please clarify.

We apologize for it. Indeed, the description of the ELISA assay for detection of IgG1 and IgE was confusing. In brief, this is not a sandwich ELISA. The plate is coated with OVA (not with IgG1 or IgE antibodies, as one could understand by how the previous description was written). Next, serum of OVA-induced allergic mice was incubated with the OVA-coated plate, followed by another incubation step with antibodies against IgG1 or IgE. There is no need for antibody enrichment, because the serum of OVA-allergic mice contained OVA-specific antibodies, that bounded the OVA coated in the well. We re-wrote this section, and we hope this is now more clear.

**Reviewer #2:**

This is a complex study (depicted in 4 dense Figures) which focuses on understanding how a major schistosome egg protein, Omega-1, suppresses the allergic airway inflammation (AAI). Experimentally it is a strong study, but the data are poorly presented and described. There are too many turns and twist with unnecessary data that asks from a reader to decipher which information is important and which is not.

*Required experiment*

The observation that immunization (pre-treatment) with Omega-1 triggers a significant IFN-gamma response was not explored by the authors as a possible underlying mechanism of the immunomodulatory effect of Omega-1 on AAI, especially since the IFN-gamma response was dependent on the intact RNAse-activity of Omega-1, which could explain decreased eosinophil recruitment in lungs of OVA/alum-primed animals.

The authors should address the role of IFN-gamma induced by Omega-1 as a factor that mediates suppression of AAI.

We appreciate reviewer’s suggestion about the role of IFNg as a putative underlying mechanism of the immunomodulatory effect of Omega-1. We aimed to address the role of IFNg through applying the full OVA/alum-AAI model with Omega-1 in IFNgR KO mice.

Since we did not have enough protein left from the recombinant Omega-1 batch we used for most of the experiments reported in the manuscript, our collaborator from the University of Wageningen produced a new batch for us. However, the new batch turned out not being optimal. This batch had significantly lower concentration compared to the previous ones we used before (~1 mg/mL vs ~5 mg/mL) and the 2 experiments we performed with this batch showed different results from the ones reported in the manuscript (>10 individual experiments). (1) Analysis of OVA+ cDC2s & CCR7+ cDC2s the PEC following omega-1 injection, showing no difference compared to the OVA control group **(Figure 1A below)**; and (2) the full allergy model in WT and IFNgR KO mice, showing no inhibition of BAL eosinophilia by omega-1 **(Figure 1B)** nor reduced Th2 cytokines in medLNs **(Fig 1C)** in the WT mice. Due to inconsistencies observed with this batch of Omega-1, we cannot properly address the role of IFNg now. This would require the production of a new batch of recombinant Omega-1, and further breeding of IFNgR-KO mice, which would cause delays of another few months, grossly exceeding the deadline for resubmission of the manuscript.

As an alternative, we now addressed the putative role for IFNg in our model in the discussion (line 332), commenting on the high levels of IFNg in the PEC and the medLNs. Of note, the PEC of omega-1 treated mice contains more cells and when calculating the cytokine production on a per cell basis **(Fig. Supplementary 3L-M)**, production levels are more similar between the groups.

**Figure 1** – Evaluation of the role of IFNg in Omega-1-induced protection against AAI. Mice were pre-treated with 50 ug ω1 or Δω1 diluted in PBS on day 0 and 7. OVA sensitization was induced by 10 μg OVA-AF647 intraperitoneal (i.p.) injected in alum adjuvant on day 12 followed by sacrifice 24 hours later **(A).** Mice were pre-treated with 50 ug ω1 or Δω1 diluted in PBS on day 0 and 7. OVA sensitization was induced by 10 μg OVA intraperitoneal (i.p.) injected in alum adjuvant (2 mg/mL on day 12 and 19). Seven days after the last injection, mice were challenged for 3 consecutive days by intranasal (i.n.) administration of 50 μg OVA in PBS. Mice were sacrificed 24 hours after the last challenge **(B;C)**. **(A)** Frequency of OVA^+^cDCs (left) and CCR7^+^ cDC2s (right) from the peritoneal lavage of treated mice. **(B)** Total cell counts (left), number (middle) and frequency of eosinophils from bronchioalveolar lavage (BAL). **(C)** Frequency of cytokine-producing cells measured in the mdLn of AAI mice by flow cytometry following polyclonal restimulation. Mean ± SEM are indicated in the graphs. One-way Anova with Tukey HSD post-test **(A)** or Two-way Anova with Tukey HSD post-test **(B;C)** was used to assess statistically significant differences; *p < 0.05, **p < 0.01, ***p < 0.001, ****p<0.0001.

*Comments*

Fig. 2 describes the analysis of lung DC (Omega-1 pre-treatment increases lung cDC1 frequencies and reduces lung cDC2 activation), although the effects observed are independent of RNAse activity of Omega-1 and, as such, appears to be less relevant to this study because Omega-1-mediated suppression of AAI is dependent on its RNAse activity. Indeed, the results in this figure are not even mentioned in the Abstract.

We appreciate the comments from the reviewer; we think that these results are still interesting, but agree they are less crucial for this study. We decided to focus in the abstract on the results that were more relevant to the message we want to convey.

In several instances, a more rigorous interpretation of the data may be required: E.g. “except BAL macrophages and lung alveolar macrophages, that were reduced in OVA/alum mice but increased in ω1-treated mice (Fig. 1C)” (line 82). However, BAL macrophages in Omega-1-treated mice were also significantly and dramatically decreased, but just slightly less than in the only OVA/alum-treated group. Moreover, there are no significant changes in lung cell composition based on Fig 1B, except for a decreased number of eosinophils (as stated in Abstract).

We agree with reviewer’s point, and to strength the data from the BAL analysis we included more data points to Figure 1C from a previous experiment. Because cell numbers were relatively different amongst the experiments, the distribution of the data points was not normal anymore, and we performed statistical analysis with Kruskal-Wallis and Dunn’s post-test correction. This approach highlights differences that before were marginal. With the addition of a second experiment, BAL macrophages are still reduced in omega-1 treated mice, but it is more clear now that Omega-1 is not *increasing* the number of macrophages in the BAL, but rather preventing the OVA/alum-induced decrease (compared to the PBS-treated mice), and we have adjusted this conclusion now in the results (line 98). This is supported by the significant difference observed in macrophage numbers between OVA/alum and OVA/alum + omega-1 groups in the BAL. Additionally, to save space in the figure (for the incorporation of new data – Figure 1J) , we combined the main immune markers in one graphic, but for Reviewer’s view, we are displaying here the data of BAL macrophages only (**Figure 2** in this point by point response).

**Figure 2.** – Cell number of BAL macrophages.

Regarding the lung composition, we should apologize for a mistake made during the cell number calculation earlier. Some immune populations displayed a trend to be increased by OVA with omega-1 preventing this increase. However, the differences did not reach significant statistical values at that time. After correction of the cells number, we now show in the lungs a similar phenotype as observed in the BAL. OVA/alum induced a shift in the macrophage composition, with reduced alveolar macrophages and increases in both CD11c+ and CD11c- interstitial macrophages. This shift was prevented by omega-1 pre-treatment. Concordantly, omega-1 also prevented the OVA/alum-induced increase in neutrophils.

Additionally, we carefully looked at the composition of interstitial macrophages, and separated these cells on the basis of CD11c expression. Even though both CD11c^+^ and CD11c^-^ interstitial macrophages were increased by OVA/alum, this increase seems to be more pronounced in the CD11c^+^ population. Interestingly, omega-1 prevented the increase of both interstitial macrophage population. We now included this in the results and attempted to better describe the changes in cellular composition in BAL and lungs of allergic mice.

It is not clear how the authors reconcile their description of Omega-1 as a “general adjuvant” (Line 88), based on induction of Th2 cytokine by Omega-1 in mice that were immunized only with OVA and its immunosuppressive effect of Omega-1 on OVA-induced AAI.

In hind sight, this is indeed confusing. Even though we have evidence that omega-1 can prime human DCs to drive polarized type 2 responses (22966004, 19635864), one would expect a similar type 2 induction in ex vivo restimulated mdLns cells from PBS- or OVA/Alum-treated mice. Since this is not the case, this may not represent the full explanation. Therefore we have removed the description of the ‘general adjuvant effect’ and rewrote the sentence.

It now reads like: “Additionally, ω1 administration (but not Δω1) reduced Th1- and Th2-associated cytokines in BAL fluid **(Figure 1D)** and OVA-specific Th2-associated cytokines in stimulated medLn cells **(Figure 1E)**; contrary, ω1-specific Th2-associated cytokines were increased, confirming its prior described immune function **(Figure 1F)**^21^.”

Focusing on very early events after OVA/alum immunization (day 1 after 1^st^ injection) at the site of injection and draining mediastinal nodes (mLN), authors show that the frequency of cDC2 that have picked-up OVA is lower in animals treated with Omega-1. However, since they also show a significant increase in the number of cDC2 in the same animals, the authors need to show the absolute number of OVA+ cDC2 in different experimental groups to make a convincing statement.

This is a fair point raised by the reviewer. We calculated cell numbers of OVA^+^ DCs and we observed a trend towards reduced number of OVA+ DCs in the mdLn of omega-1 pre-treated mice. Due to the differences in overall Ln cellularity the spread of the dots was largely impacting the statistical calculations, which prevented the analysis to reach statistical significance. We have now included this data in Figure Supp 4C and in the results section (line 201).

As a punch line, the authors perform a migratory assay showing that cDC2 from Omega-1-pretreated mice display (~20%) decreased ability to migrate in an in vitro assay in response CCL19. The degree to which decreased DC expression of CCR7 or F-actin (need to be brought up in Abstract and main Figure) in mice primed with Omega-1 contributes to “slower” migration is unclear at present.

We appreciate reviewer’s fair point and have tried to address this in the discussion. Indeed, omega-1 has been show to affect the spreading and the morphology of monocyte-derived dendritic cells (19635859), but SEA-treated DCs were faster than immature DCs, when moving through fibronectin-coated plates (30120122). Whether w1 itself has the same effect of SEA (a mixture in which omega-1 is abundantly present) in terms of DCs motility, or whether SEA and potentially w1 have the same effect *in vivo* as they have *in vitro,* is still subject for debate. We know that F-actin is important for cell motility (21940796) and to the formation of the immunological synapse between DC and T cells (26590149),while w1 has also been shown to impair that formation of DC-T cell conjugates (19635859). Our observation that w1-treated PEC DCs display reduced F-actin would be in line with the importance of F-actin to both migration and T cell priming by DCs. To what extend reduced CCR7 expression, reduced F-actin or a combination of both factors contributes to the reduced motility and/or reduced migration of PEC DCs is still an open question. Nevertheless, we argue that CCR7 expression may plays a bigger role in our model compared to F-actin because the *in vitro* impaired migratory capacity of DCs, and the CCR7 expression were both dependent on Omega-1 RNAse activity, whereas the impaired F-actin formation observed in Omega-1 immunized mice was not (F-actin was still reduced in mutant-treated mice, while the migratory capacity of DCs was not impaired). This could suggest that the reduced migratory capacity of PEC cDC2s is more associated with their reduced CCR7 expression rather than their reduced F-actin staining. We included this in our discussion section (line 292).
